# Reduced cortical brain perfusion following COVID-19 infection: impact of COVID-19 severity and relation to memory performance

**DOI:** 10.3389/fnhum.2026.1575787

**Published:** 2026-04-10

**Authors:** Justin M. Palmer, Stephanie Matijevic, Chidi Ugonna, Angelica Galdamez-Avila, Aidan Rhodes, Nan-kuei Chen, Juan C. Arias, Dianne Patterson, Danny J. J. Wang, Lee Ryan

**Affiliations:** 1 The University of Arizona, Tucson, AZ, United States; 2 Arizona Alzheimer's Consortium, Phoenix, AZ, United States; 3 Division of Vascular Surgery, The University of Arizona, Tucson, AZ, United States; 4 Laboratory of FMRI Technology (LOFT), Mark and Mary Stevens Neuroimaging and Informatics Institute, University of Southern California, Los Angeles, CA, United States; 5 Evelyn F. McKnight Brain Institute, Tucson, AZ, United States

**Keywords:** acute respiratory distress syndrome, ASL, COVID-19, diffusion, episodic memory, pattern separation, perfusion

## Abstract

**Introduction:**

Changes to brain perfusion may be contributing to long-term cognitive dysfunction following COVID-19 infection. Lower brain perfusion beyond the acute phase of infection, as measured by perfusion MRI, has been observed in both hospitalized and non-hospitalized cases. Additionally, the hippocampus has been previously shown to be particularly sensitive to changes in blood flow. Therefore, the hippocampus may be vulnerable to perfusion changes associated with COVID-19, leading to long-term cognitive sequalae. The literature suggest that this may be particularly true among those with severe respiratory symptoms. However, the relationship between COVID-19 respiratory severity, perfusion, and cognitive dysfunction has yet to be thoroughly explored, especially after longer recovery times.

**Methods:**

Seventy-eight participants completed a neuroimaging session and two memory tests, the Mnemonic Similarity Task (MST) and Face-Name Associative Memory Exam (FNAME), tasks known to be mediated by the hippocampus. Participants were divided into four groups based on the severity of self-reported respiratory symptoms experienced during the acute infection: controls without a reported diagnosis of COVID-19, those with COVID-19 and without respiratory symptoms, those with COVID-19 and respiratory symptoms, and hospitalized participants with respiratory symptoms from COVID-19. On average, participants were 13 months from initial infection. Perfusion was measured with pseudo-continuous arterial spin labeled (pCASL) MRI. Total cortical gray and white matter perfusion, as well as gray matter perfusion in the territories of the anterior, middle, and posterior cerebral arteries were obtained using a mask adapted from previous studies.

**Results:**

The hospitalized group demonstrated lower total gray matter cortical perfusion compared to all groups, *t*'s > 1.74, *p*'s < 0.05. White matter perfusion showed no differences between groups. A similar pattern was observed within each arterial territory. Out of all memory measures, only pattern separation from the MST was related to gray matter perfusion, *r* = 0.30, *p* < 0.01, even after controlling for age and education.

**Discussion:**

Results provide evidence for global perfusion dysfunction among hospitalized participants with severe respiratory symptoms that persists for more than a year after COVID-19 infection. Additionally, there was a relationship between perfusion and pattern separation performance among all participants, suggesting that poorer perfusion may underlie some of the long-term COVID-19 cognitive symptoms.

## 1 Introduction

Cognitive difficulties are common long-term symptoms reported following COVID-19 infection, predominantly in areas of memory and attention (Ceban et al., 2022; Søraas et al., 2021; Tavares-Júnior et al., 2022; Hellmuth et al., 2021; Frontera et al., 2021; Malheiro et al., 2024), and can persist for several months and up to 11 months from infection (Voruz et al., 2022; Becker et al., 2021; Díez-Cirarda et al., 2023a). Importantly, cognitive symptoms following infection are not just observed among severe cases of COVID-19, but also among non-hospitalized cases (Tenforde et al., 2020; Michelen et al., 2021; Moreno-Pérez et al., 2021; Bell et al., 2021; Peghin et al., 2021; Del Corral et al., 2024). Those with severe respiratory symptoms such as acute respiratory distress syndrome (ARDS) during acute COVID-19 infection may be at a higher risk for cognitive dysfunction following recovery (Dondaine et al., 2022; Helms et al., 2020; Ermis et al., 2021; Beaud et al., 2021; Jaquet et al., 2022). For example, one study evaluated COVID-19 participants with ARDS at 3, 6, and 12 months following infection (Latronico et al., 2022). Many individuals were shown to experience improvements in MoCA scores over time; however, 27% and 16% of their participants still scored below the standard cutoff of 26 at 6 and 12 months, respectively, suggesting that some, but not all, COVID-19 participants with ARDS still experience some level of cognitive difficulty a year from infection (Latronico et al., 2022).

However, very few studies go beyond using the MoCA to assess cognition. Given that this instrument was designed to detect the presence of dementia, it is relatively insensitive to measure subtle cognitive impairment. Studies evaluating COVID-19 participants with ARDS using more thorough cognitive testing have previously shown worse performance on verbal memory tests compared to COVID-19 patients without ARDS 4 months following infection (Ferrucci et al., 2021) and on tests of executive function 6 months from infection (Duindam et al., 2022). Surprisingly, a recent systematic review described that many studies find no relationship between memory performance following recovery and overall severity of illness during the acute stage (Llana et al., 2024). However, one notable exception was a relationship between markers of acute respiratory failure and verbal memory performance (Llana et al., 2024). Taken together, respiratory symptoms appear to be an important factor when understanding long-term cognitive functioning following infection, but few studies include participants ranging in respiratory symptom severity while also utilizing thorough cognitive tests at longer timepoints from recovery.

The mechanisms leading to cognitive difficulties following infection are still poorly understood. Neurovascular dysfunction during acute infection appears to be an important mechanism leading to long-term cognitive sequelae (Lyra E. Silva et al., 2022; Vanderheiden and Klein, 2022; Miners et al., 2020). Decreased perfusion was observed in several early studies of COVID-19 cases using perfusion MRI (Soldatelli et al., 2020; Helms et al., 2020), especially among the most severe cases (Chougar et al., 2020; Ardellier et al., 2023). Poor cerebral blood flow (CBF) following COVID-19 has been shown to extend beyond the acute infection among hospitalized and non-hospitalized cases (Kim et al., 2023; Ajčević et al., 2023), with some studies indicating that the severity of symptoms is a risk factor for poorer perfusion after infection (Qin et al., 2021). However, the relationship between perfusion and cognitive performance following COVID-19 is not as clear. The few studies that have investigated perfusion and cognition are mixed and limited by the use of cognitive screeners and various recovery times (Hosp et al., 2021; Ajčević et al., 2023). Additionally, even fewer studies focus on perfusion within the hippocampus, a critical area for learning and memory that is particularly sensitive to changes in blood supply (Fujioka et al., 2000; Masayuki et al., 1996; Petito et al., 1987; Fotuhi et al., 2012; Laosiripisan et al., 2015) and following hypoxia (see Lana et al., 2020 for review). A recent study focusing on the effects on the hippocampus from COVID-19 infection found smaller hippocampal volumes and lower perfusion among those diagnosed with post-COVID syndrome (PCS; defined as persisting symptoms for at least 12 weeks from infection) and reporting subjective cognitive complaints 11 months from infection compared to healthy controls matched on age and sex who never had COVID-19 (Díez-Cirarda et al., 2023b). Reduced perfusion within the hippocampus was correlated with measures of visual and verbal memory (Díez-Cirarda et al., 2023b). However, global perfusion or perfusion in other areas were not assessed, making it unclear if these results reflected specific changes to the hippocampus or related to more global changes in perfusion following infection.

Poorer cognitive functioning following COVID-19 has also been linked to white matter damage in hospitalized cases (Lu et al., 2020; Raman et al., 2021) and non-hospitalized cases of COVID-19 (Scardua-Silva et al., 2024) using diffusion-weighted imaging several months from infection. However, the relationship between cognitive performance and white matter damage does not appear to persist in studies that use recovery times beyond 10 months (Díez-Cirarda et al., 2023a; Huang et al., 2022; Tian et al., 2022). A comprehensive study that includes both perfusion and diffusion outcomes among participants with a range of respiratory symptoms can better assess the potential of multiple mechanisms contributing to cognitive performance.

Overall, most of the early studies evaluating perfusion outcomes from COVID-19 infection lack large sample sizes and matched controls, making it difficult to compare results across studies. Studies with longer recovery times are beginning to emerge and suggest long-term perfusion dysfunction may be present among both hospitalized and non-hospitalized cases. These findings are in contrast to studies that evaluate white matter integrity, which suggest that damage to the white matter may recover sooner than perfusion changes. The prolonged perfusion disruption may have particular implications for hippocampal functioning and cognitive sequelae following infection. Therefore, a study evaluating the relationship between perfusion and cognitive performance on tests designed to rely on the integrity of the hippocampus is needed.

### 1.1 Present study

The present study investigated how the severity of respiratory symptoms during COVID-19 infection related to global perfusion and perfusion within the specific arterial territories. Importantly, we focused on recruiting participants over a year from infection, on average. We used pseudo-continuous arterial-spin label (pCASL) imaging as a non-invasive method to quantify CBF in absolute units, allowing for direct comparison across individuals and groups (Alsop et al., 2015). Changes to perfusion from COVID-19 may not be the only contributor to cognitive dysfunction following infection. Though not the focus of the study, we also assessed disruption to the white matter integrity using diffusion tensor imaging in order to evaluate if white matter damage was also present among participants. Including both perfusion and diffusion measures in the present study will help clarify if long-term disruption to perfusion is contributing uniquely to cognitive dysfunction following COVID-19 infection. Additionally, we examined the relationship between perfusion and diffusion outcomes and cognitive functioning using sensitive tests known to rely on the hippocampus, an area that appears to be particularly sensitive to disrupted perfusion. Participants ranging in respiratory symptom severity were compared to a carefully matched control group who did not report having COVID-19.

## 2 Materials and methods

### 2.1 Participants

Most participants were recruited via advertisements in the newspaper and on social media. Others were approached at Banner Health's Pulmonology Clinic in Tucson, Arizona, recruited from our database and ongoing collaborations, or from community talks. Interested participants were screened for eligibility over the phone. Eligible participants were those with no history of a prior diagnosis of a neurological disorder, stroke, dementia, or psychotic illness. Eighty participants underwent a comprehensive cognitive assessment battery and a complete magnetic resonance imaging (MRI) session. All images underwent manual inspection to control for quality and absence of gross abnormalities, resulting in 78 usable scans for subsequent analyses. COVID-19 severity was determined by the presence or absence of respiratory symptoms using a brief COVID-19 survey through Qualtrics™. Participants were shown a list of common symptoms and instructed to check any that they experienced during their infection. Symptoms listed included: sore throat, cough, fever, shortness of breath/labored breathing, vomiting, nausea, headache, diarrhea, confusion, exhaustion/excessive sleepiness, loss of taste, loss of smell, muscle/body aches, congestion/runny nose, chest pain/pressure, bluish lips/face/toes, or hallucinations. Ongoing fatigue and cognitive changes since infection were also reported. Responses from the COVID-19 survey were used to classify individuals into four groups based on the severity of respiratory symptoms they experienced at the time of infection (referred to as COVID-19 Severity Groups). Group 1 served as our control group, who did not report a diagnosis of COVID-19 or experience COVID-19-related symptoms since 2020 (*n* = 18). Group 2 were participants who reported a diagnosis of COVID-19 without respiratory symptoms (*n* = 26). Group 3 were participants who reported a diagnosis with COVID-19 with reported respiratory symptoms, classified as labored breathing/shortness of breath or chest pain/pressure (*n* = 26). Group 4 were participants who were hospitalized with respiratory symptoms from COVID-19 (*n* = 8). Group demographics can be found in Table 1. Groups did not differ on age, *F*_(3, 77)_ = 1.9, ns, or education, *F*_(3, 77)_ = 1.8, ns. Fifty-two participants were able to provide the date they were diagnosed. Time from diagnosis to test was not significant between groups, *F*_(2, 50)_ = 2.25, ns. Common cardiovascular factors, including heart disease, high blood pressure, high cholesterol, and diabetes were not different between groups. Written, informed consent was collected in accordance with the University of Arizona's Institutional Review Board.

**Table 1:** Average age, average years of education, and sex within each COVID-19 severity group.

Group	Average age (SD)	Average education (SD)	Sex (F:M)	Time from diagnosis to test (Months)
Controls (*N* = 18)	61.7 (10.3)	17.3 (1.9)	14:4	
No respiratory symptoms (*N* = 26)	57.7 (11.6)	17.0 (2.6)	18:8	10.36 (7.16)
With respiratory symptoms (*N* = 26)	53.9 (11.0)	16.2 (1.9)	20:6	15.83 (11.01)
Hospitalized (*N* = 8)	60.4 (11.2)	15.5 (2.1)	6:2	15.71 (7.83)

No differences in age or education existed between groups [age: *F*_(3, 77)_ = 1.9; education: *F*_(3, 77)_ = 1.8, ns].

### 2.2 Hippocampally-mediated tasks

The MST was used to assess mnemonic discrimination as a common proxy for pattern separation, which has been demonstrated to be dependent on the hippocampus (Yassa and Stark, 2011; Yassa et al., 2011; Stark and Stark, 2017). In the first phase of the task, participants were shown 128 common objects on a white background, one at a time, and identified if the object is found indoors or outdoors. Participants were then given a surprise memory test where they were shown objects again, one at a time, and identified identical and perceptually similar objects from the first phase, as well as novel objects never seen before. Sixty-four objects were identical to the objects in the first phase, 64 objects were perceptually similar to the objects in the first phase, and 64 objects were novel. Primary dependent measures included object recognition as the proportion of correct responses for old objects corrected for false alarms (i.e., the proportion of novel objects identified as old) and pattern separation as the proportion of correct responses for similar objects corrected for false alarms (i.e., the proportion of novel objects identified as similar). Two participants mixed up the response buttons on the MST task and were excluded from the cognitive analyses.

FNAME provides another measure of memory function dependent on the hippocampus (Papp et al., 2014; Sperling et al., 2003). In this task, 12 different faces were shown one at a time using PowerPoint, each with a different name and occupation shown below the face. Each face was shown for 8 s, and participants were asked to read the name and occupation of each face aloud. Participants were given two learning and recall trials. Following, participants were given a distraction task where they were shown the faces of 12 different celebrities and asked to identify the name and occupation of each famous person, one at a time. All 12 previously learned faces were then shown again, one at a time, and participants were asked to recall the name and occupation of each presented face. Following a 30-min delay, participants were given a recognition trial of each face among two other distractor faces never seen before. Participants were asked to recall the name and occupation of that face followed by a multiple-choice trial for that name and occupation. Primary dependent measures included total correct names recalled across all trials (maximum score of 48) and total correct occupations recalled across all trials (maximum score of 48).

### 2.3 MRI acquisition and preprocessing

All MRI scans were performed on the University of Arizona's 3T Siemens Skyra scanner with a 32-channel head coil. The MRI protocol included a 3D T1-weighted (T1w) MPRAGE sequence (1.0 mm isotropic voxel size, TR = 2,300 ms, TE = 2.98 ms, and FOV=256 mm), a pCASL sequence with a single post-label delay (PLD), a calibration image, and six control-label pairs (TR = 5,000 ms, TE = 22.58 ms, PLD = 1,800 ms, label duration = 1,500 ms, 3.0 mm isotropic voxels, FOV=192 mm, with background suppression), and an echo-planar-multishell diffusion sequence to collect diffusion-weighted images in 30 directions (*b*-values = 500–2,000 s/mm^2^, 54 slices, voxel size = 2.5 mm^3^, TR = 4,200 ms, TE = 9.7 ms, FOV=256 mm^2^).

Arterial spin labeling uses water in the arterial blood as an endogenous tracer by labeling inflowing blood in the neck and waiting for the labeled blood to perfuse the brain prior to imaging. This labeled image is followed by a control image of the brain without labeling, allowing for the subtraction between the label and control pair to reflect the contribution of signal from perfusion in arbitrary units. Absolute units (ml/100 g/min) are calculated using a calibration, proton-density weighted image (also called the M0) in order to quantify the magnetization of the arterial blood (Chappell et al., 2018; Clement et al., 2022). T1w images were defaced using pydeface, and subsequently skull-stripped using BET with a fractional intensity threshold of 0.5. Fsl_anat was used for T1w anatomical segmentation and registration to MNI152 standard space. Preprocessing of ASL data was carried out using oxford_asl in FMRIB Software Library (FSL, www.fmrib.ox.ac.uk/fsl), which included motion correction using MCFLIRT (Jenkinson et al., 2002) and distortion correction using field maps. Oxford_asl performed linear registration of the perfusion-weighted image to the anatomical image. Quantification of CBF in absolute values was also carried out with oxford_asl, using an inversion efficiency of 0.85 and assuming T1 values for tissue and arterial blood as 1.3 and 1.65 s, respectively (Alsop et al., 2015), with CSF as the reference tissue. Spatial regularization was also used to correct for noise in the final perfusion-weighted images.

A chemical shift artifact Hood et al., (1999) was observed in the phase-encoding direction of the M0 and the ASL acquisitions (control and label) for each subject with varying levels of prominence. The artifact is manifested as a bright signal corresponding to the spatial misalignment of fat located in the scalp and primarily located in the posterior of the brain (Supplementary Figure 2). The artifact was not completely eliminated after control and label subtraction to produce the perfusion image. A mask of the location of the artifact for each subject was created by first using the function *make_scalp_surfaces* from the open-source application MNE-python (Gramfort et al., 2013) to create an outer skull surface from the Freesurfer (Fischl, 2012) surfaces created from the subject's T1w acquisition and converting this outer skull surface to a volume mask. The approximate expected spatial shift of the scalp surface was calculated as 18 mm based on the ASL's bandwidth per pixel in the phase encoding direction and the voxel mask was translated by this distance in the phase-encoding direction. To ensure conservative coverage of the chemical shift artifact for all subjects, the mask was dilated by combining individual masks calculated over a range of spatial shifts at 15, 21, and 24 mm. This combined mask was then transformed to ASL space by using affine transforms already calculated by oxford_asl above. A visual check was performed on all subjects to confirm that the chemical shift artifact was indeed correctly encompassed by the mask. This mask was then subsequently used to ignore potentially corrupted voxels during the statistical analysis.

ASL data is particularly sensitive to partial volume effects (PVE) given the low spatial resolution and the large differences between perfusion in the gray and white matter (Clement et al., 2022). Gray matter perfusion, on average, is approximately three times higher than the white matter perfusion (Chappell et al., 2018), so careful consideration must be taken when calculating perfusion across the whole brain and within ROIs. However, no consensus has been established to correct for PVE, despite many studies addressing this concern (see Chappell et al., 2021 for review). Thresholding the perfusion maps across the whole brain to only include voxels with a high probability of gray matter (or white matter) based on the T1 partial volume estimates is one approach (Petr et al., 2018). Unfortunately, using conservative thresholds can leave very few voxels in the analysis, lowering the SNR and statistical power (Clement et al., 2022). Additionally, PVE can be more complicated in studies with older adult populations (Chen et al., 2011) or clinical populations with suspected brain atrophy (Johnson et al., 2005; Chen et al., 2012; Binnewijzend et al., 2013), leading to overestimates of hypoperfusion that could be attributed to the loss of brain volume, the genuine reduction of perfusion, or both (Chappell et al., 2021).

As an alternative, using partial volume correction (PVC) increases the statistical power and provides a more robust way to calculate perfusion without excluding a large number of voxels (Chappell et al., 2021). PVC estimates perfusion by modeling the signal from each tissue compartment in each voxel using partial volume estimates from a high-resolution anatomical image (Asllani et al., 2008). This approach was evaluated in a study that compared the effects of thresholding voxels and using PVC, and found better quality perfusion maps with fewer errors when using PVC (Petr et al., 2018). It is important to consider that these models for PVC rely on successful segmentation of gray and white matter for accurate perfusion estimates.

**Figure 1:**
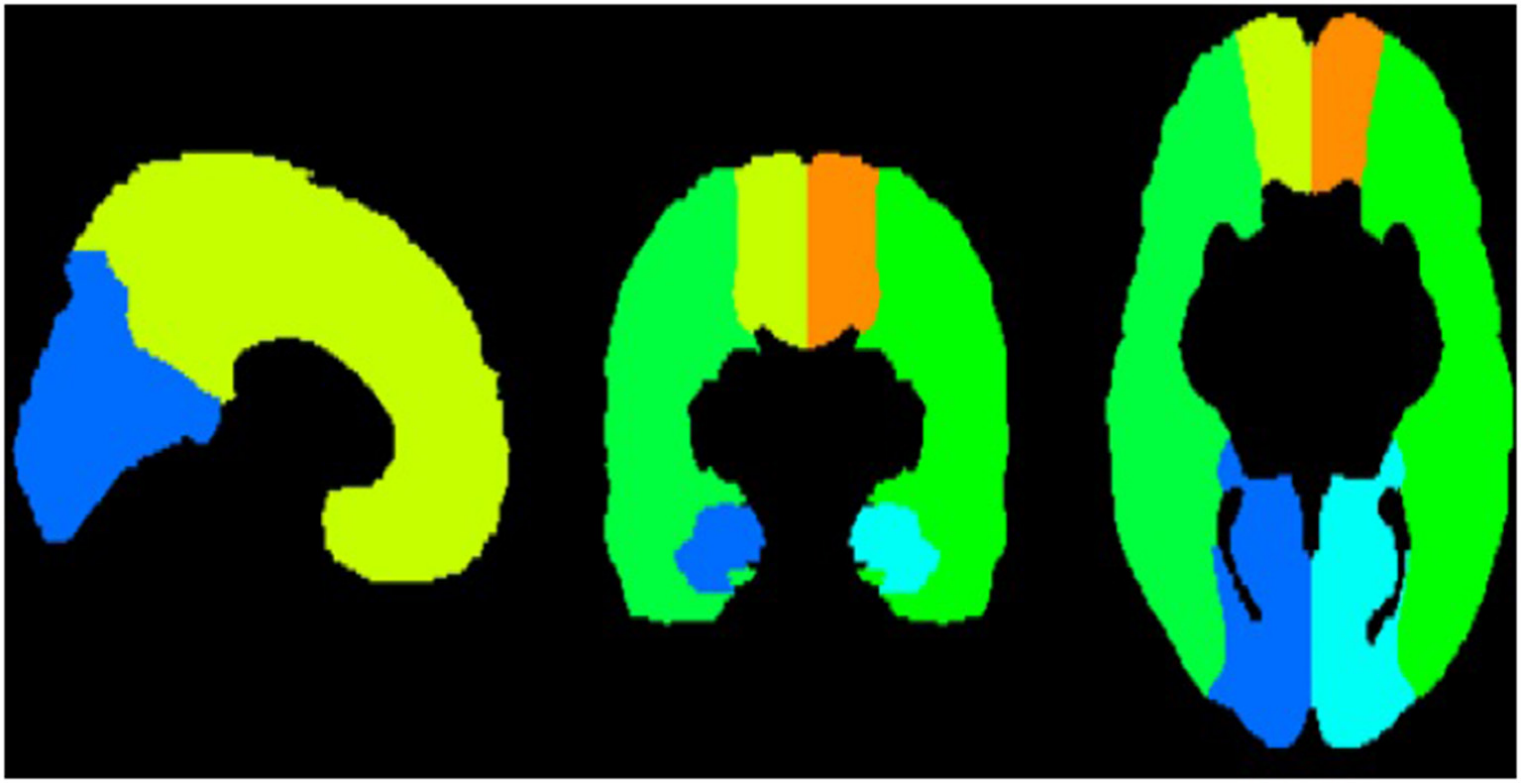
Cortical mask adapted from (Liu et al. 2023) that excludes subcortical structures and the cerebellum. ACA is depicted in orange and yellow. MCA is depicted in green. PCA is depicted in blue.

Physiological differences and the complexities of subcortical vascular supply pose another concern when looking at total brain perfusion (Chappell et al., 2023). The subcortical structures will also need a longer arterial transit time compared to cortical structures, and including them in analyses could underestimate perfusion values if the labeled blood-water has not been given sufficient time to arrive. One study acknowledged how subcortical structures are more prone to physiologic noise, which can make estimating perfusion in these areas more difficult (Chand et al., 2020). Additionally, the poor spatial resolution in areas like the striatum will substantially increase PVE and noise. To overcome this barrier, we developed a cortical mask of the three major arterial territories (ACA; MCA; PCA) adapted from Liu et al.'s (2023) Arterial Atlas, which included the following intensity values to threshold cortical areas and eliminate subcortical structures (included intensity values: 1–2, 7–14, 17–20, and 23–24; see Figure 1 and Supplementary Material for further details). A weighted sum of perfusion within each of the three arterial territories was calculated to estimate total cortical perfusion for the gray and white matter.

Diffusion preprocessing and reconstruction was performed using QSIPrep 0.18.0, which is based on Nipype 1.8.6 (Gorgolewski et al., 2011; RRID:SCR_002502). Any images with a *b*-value less than 100 s/mm^2^ were treated as a *b* = 0 image. MP-PCA denoising as implemented in MRtrix3's dwidenoise (Veraart et al., 2016) was applied with a 5-voxel window. After MP-PCA, the mean intensity of the DWI series was adjusted so the mean intensity of the *b* = 0 images matched across each separate DWI scanning sequence. B1 field inhomogeneity was corrected using dwibiascorrect from MRtrix3 with the N4 algorithm (Tustison et al., 2010) after corrected images were resampled. FSL (version 6.0.5.1:57b01774) eddy was used for head motion correction and eddy current correction (Andersson and Sotiropoulos, 2016). TOPUP was used for susceptibility distortion correction. DTIfit was used to model tensors at each voxel to produce FA maps for each participant. Each participant's FA map was non-linearly registered to a 2 mm FA map in MNI152 space. Global FA metrics were calculated using a threshold of 0.25 on each participant's total map as well as mean, radial, and axial diffusivities (MD; RD; AD).

### 2.4 Plan of analysis and hypotheses

The two primary measures from the MST (object recognition and pattern separation) and the two primary measures from the FNAME (FNN and FNO) were analyzed using separate one-way ANOVAs. *Post-hoc* group differences were analyzed using LSD tests. Additionally, to assess for effects of increasing severity of respiratory symptoms within the three COVID-19 groups only, the controls were removed and analyzed in separate ANOVAs. We hypothesized that all COVID-19 groups will perform worse on pattern separation and FNN relative to controls, measures most sensitive to hippocampal functioning, while no differences are hypothesized on measures of object recognition and FNO.

Similarly, we also hypothesized that all COVID-19 groups will have lower total cortical gray matter perfusion compared to controls who did not report having COVID-19 using independent samples *t*-tests. Additionally, the COVID-19 groups will demonstrate a step-wise decrement in cortical gray matter perfusion with increasing respiratory severity using one-way ANOVAs and independent samples *t*-tests. Analyses within each arterial territory (ACA, MCA, and PCA) were conducted in the same way to assess for global and regional perfusion outcomes. Correlations between gray and white matter cortical perfusion and the four memory outcomes (object recognition, pattern separation, FNO, and FNN) were also conducted, with the strongest relationship predicted to be between gray matter perfusion and pattern separation and FNN. Follow-up univariate general linear models (GLM) will be used to analyze significant correlations to assess for main effects of cortical perfusion, COVID-19 Severity Group, and their interaction, while controlling for age.

Independent samples *t*-tests were used to compare the controls who did not report having COVID-19 to each of the three COVID-19 groups to assess for mean differences in global diffusion measures (FA, MD, AD, and RD). Consistent with the perfusion analyses, one-way ANOVAs excluding the controls were conducted to assess global measures of diffusion and COVID-19 severity. Finally, correlations between global diffusion measures, age, and primary cognitive measures were conducted.

## 3 Results

### 3.1 Cognitive tests

Performance on the hippocampally-based tasks (MST and FNAME) is displayed in Table 2. Two people were excluded due to having abnormally high false alarm rates on the MST because they mixed up the keys on the task, resulting in 76 people included for the cognitive analyses. Average object recognition for each group on the MST was the proportion of correctly identified old objects after subtracting out false alarms (i.e., the proportion of novel objects identified as old). Average pattern separation for each group on the MST was the proportion of correctly identified similar objects after subtracting out false alarms (i.e., the proportion of novel objects identified as similar). One-way ANOVAs indicated that groups performed no differently at recognizing old objects, *F*_(3, 75)_ = 1.27, ns, or correctly identifying similar objects, *F*_(3, 75)_ = 0.58, ns. No group differences in the total recall of occupations, *F*_(3, 75)_ = 0.02, ns, or total correct names, *F*_(3, 75)_ = 0.78, ns, were observed.

**Table 2:** Descriptive statistics for the four primary outcome measures on hippocampally-mediated tasks: object recognition, pattern separation, total correct occupations, and total correct names.

Hippocampal tasks	Mean	SD	Minimum	Maximum
*MST*				
**Object recognition**				
Controls	0.85	0.09	0.63	0.98
No respiratory	0.89	0.07	0.75	0.97
Respiratory	0.88	0.07	0.70	0.98
Hospitalized	0.84	0.06	0.75	0.92
**Pattern separation**				
Controls	0.26	0.16	0.04	0.56
No respiratory	0.30	0.20	0.00	0.62
Respiratory	0.33	0.21	−0.08	0.69
Hospitalized	0.25	0.19	0.09	0.65
*FNAME*				
**Occupations**				
Controls	41.06	7.16	27	48
No respiratory	40.56	8.50	16	48
Respiratory	40.42	7.85	13	47
Hospitalized	40.63	6.93	26	47
**Names**				
Controls	33.12	11.30	6	45
No respiratory	29.28	11.07	7	46
Respiratory	30.31	9.74	8	42
Hospitalized	26.75	10.28	14	40

A one-way ANOVA indicated no group differences on object recognition, *F*_(3, 75)_ = 1.27, ns, or pattern separation, *F*_(3, 75)_ = 0.58. Additionally, groups performed no differently on recalling occupations, *F*_(3, 75)_ = 0.02, ns, or total correct names, *F*_(3, 75)_ = 0.78, ns.

### 3.2 Group differences between cortical gray and white matter perfusion

**Figure 2:**
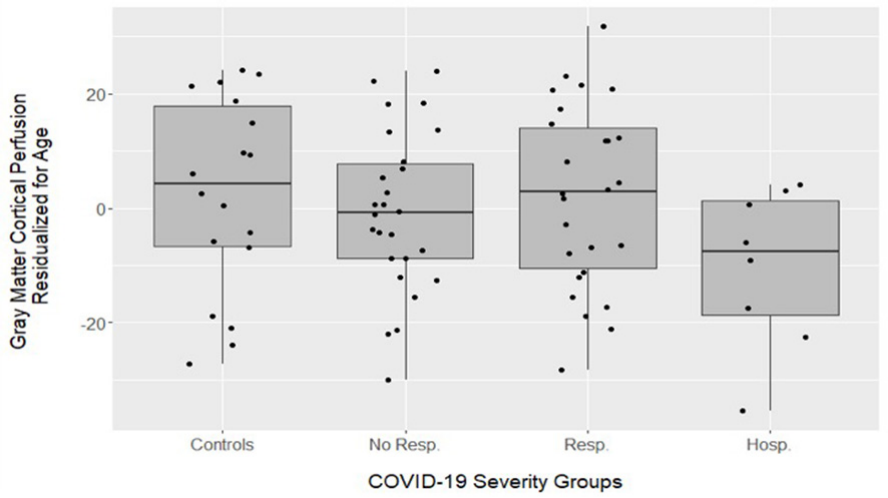
Box plot displaying cortical gray matter perfusion (residualized for age) between the COVID-19 severity groups. The hospitalized group had significantly lower perfusion than all other groups [compared to: controls *t*_(24)_ = 1.88, *p* < 0.05], those without respiratory symptoms [*t*_(32)_ = 1.74, *p* < 0.05] and those with respiratory symptoms [*t*_(32)_ = 2.20, *p* < 0.05].

One-sided independent samples *t*-tests were used to compare the cortical gray matter perfusion between controls who did not report a diagnosis of COVID-19 and each of the three COVID-19 groups, see Table 3. The hospitalized group was the only group with significantly lower cortical gray matter perfusion than controls, *t*_(24)_ = 1.88, *p* < 0.05, *d* = 0.80 (all other *t*'s < 1, ns) (Figure 2). Additionally, a one-way ANOVA assessing group differences in global perfusion without the control group revealed a marginal effect of severity group [*F*_(2, 59)_ = 2.52, *p* = 0.09]. Our *a priori* hypothesis of a stepwise decrement was further tested with independent samples *t*-tests. The hospitalized group had lower perfusion compared to the group without respiratory symptoms [*t*_(32)_ = 1.74, *p* < 0.05, *d* = 0.70] and the group with respiratory symptoms [*t*_(32)_ = 2.20, *p* < 0.05, *d* = 0.89]. No perfusion differences between the non-hospitalized COVID-19 groups were found, *t*_(50)_ = 0.84, ns. A *post-hoc* exploratory analysis to evaluate if those reporting ongoing cognitive changes (*n* = 18) had lower cortical gray matter perfusion compared to those that did not report cognitive changes (*n* = 42) also revealed no differences, *t*_(58)_ = 0.15, ns. Cognitive change was subjectively reported as a binary variable (yes or no), indicating if individuals noticed cognitive changes since their infection on the Qualtrics survey. No differences in total cortical white matter perfusion between controls who did not report a diagnosis of COVID-19 and any of the COVID-19 groups were found, all *t*'s < 1.47. Additionally, the cortical white matter perfusion did not differ between any of the three COVID-19 groups when the controls were excluded, all *t*'s < 1.42.

**Table 3:** Means (SD) for global and regional gray matter perfusion and white matter perfusion (mL/100 g/min) among the COVID-19 severity groups.

Group	Cortical gray matter	Cortical white matter	ACA	MCA	PCA
Controls (*N* = 18)	75.71 (16.74)	45.00 (12.77)	75.15 (18.20)	77.97 (17.97)	68.13 (15.38)
No respiratory symptoms (*N* = 26)	73.22 (14.69)	42.92 (9.38)	72.96 (15.62)	74.19 (14.19)	70.30 (17.80)
With respiratory symptoms(*N* = 26)	76.81 (16.01)	41.95 (8.81)	77.93 (16.89)	78.07 (16.64)	70.02 (15.48)
Hospitalized (*N* = 8)	63.11 (13.21)^*^	37.16 (11.97)	62.01 (16.46)^*^	65.15 (13.23)^*^	57.00 (11.52)^*^

Independent samples *t*-tests revealed the hospitalized group had lower perfusion compared to controls [*t*_(24)_ = 1.88, *p* < 0.05], those without respiratory symptoms [*t*_(32)_ = 1.74, *p* < 0.05] and those with respiratory symptoms [*t*_(32)_ = 2.20, *p* < 0.05]. Perfusion within each arterial territory among groups was also consistent with global perfusion, with the hospitalized group demonstrating the lowest perfusion based on independent samples t-tests (though the difference between the hospitalized group and the group without respiratory symptoms was marginal in the MCA). No significant differences in cortical white matter perfusion were found between any group.

### 3.3 Perfusion and hippocampally mediated tasks

**Figure 3:**
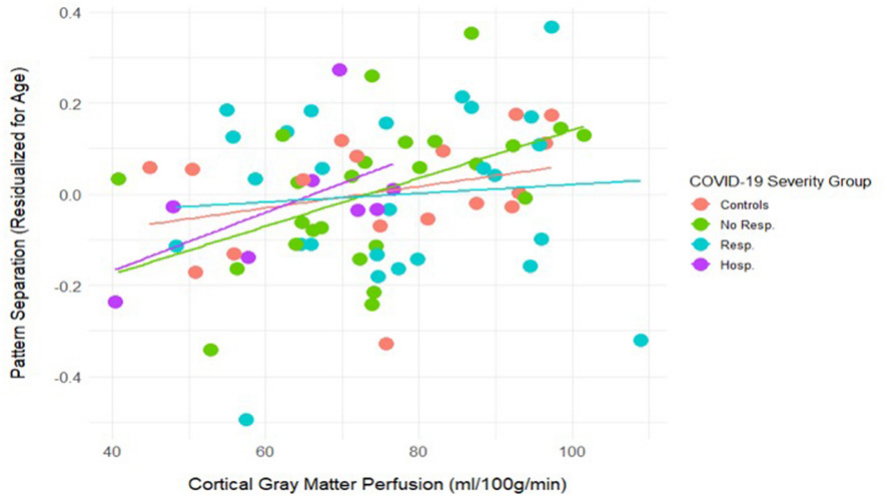
Scatter plot of cortical gray matter perfusion and pattern separation (residualized for age; *r* = 0.28, *p* < 0.05). Orange represents the controls, green represents those without respiratory symptoms, blue represents those with respiratory symptoms, and purple represents those who were hospitalized. A univariate GLM indicated a significant main effect of age [*F*_(1, 75)_ = 24.12, *p* < 0.001] and cortical gray matter perfusion [*F*_(1, 75)_ = 6.38, *p* < 0.05].

Correlations between cortical gray matter perfusion, age, object recognition, pattern separation, FNO, and FNN are displayed in Table 4. Cortical gray matter perfusion is correlated with pattern separation (*r* = 0.30, *p* < 0.01; Figure 3), but not with object recognition, FNN, FNO, or age. Because age is strongly correlated with pattern separation, partial correlations between pattern separation and cortical gray matter while controlling for age were also conducted (shown in parentheses in Table 4), and this did not change the finding (*r* = 0.28, *p* < 0.05). A univariate GLM was used to analyze the effect of cortical gray matter perfusion, COVID-19 Severity Group, and their interaction on pattern separation scores, with age as a covariate. Pattern separation scores were impacted by cortical gray matter perfusion, *F*_(1, 75)_ = 6.38, *p* < 0.05, as well as age, *F*_(1, 75)_ = 24.12, *p* < 0.001. No significant interaction was found, *F*_(1, 75)_ = 0.93, ns. White matter perfusion was not correlated with age or any cognitive measures (Table 4).

**Table 4:** Correlations between outcomes on hippocampally-dependent tasks, age, and perfusion in the cortical gray and white matter.

Outcomes	Cortical gray matter perfusion	Cortical white matter perfusion	Age
Age	−0.13	−0.01	
Object recognition	0.15	0.07	−0.31^**^
Pattern separation	0.30^**^ (0.28^*^)	0.14	−0.55^**^
FNO	−0.01	−0.03	−0.28^*^
FNN	0.01	−0.11	−0.29^*^

Partial correlations between cortical gray matter perfusion and pattern separation, controlled for age, are presented in parentheses.

^**^p < 0.01,

^*^p < 0.05.

**Figure 4:**
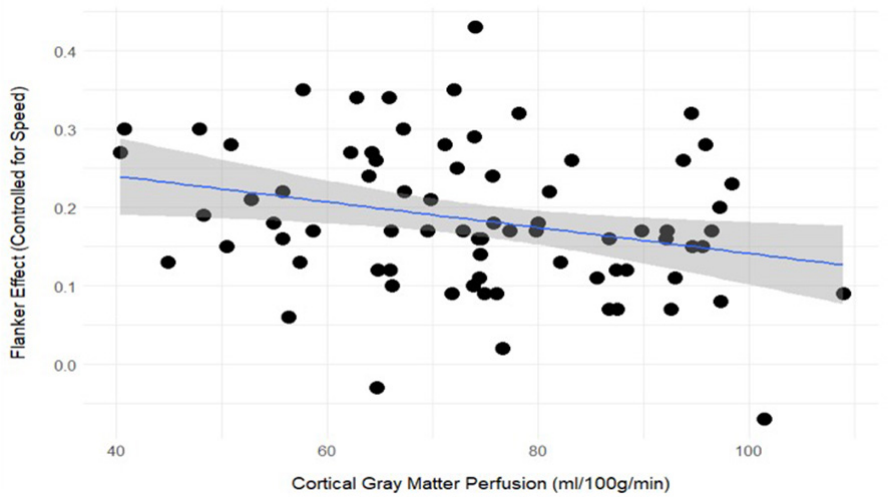
Scatter plot of Flanker effect (divided by reaction time on congruent trials) and cortical gray matter perfusion for all individuals, *r* = −0.28, *p* < 0.05.

Cortical gray matter perfusion relating to pattern separation, but not FNN, was surprising given that both tasks rely on the integrity of the hippocampus. This could indicate that other cognitive processes beyond memory are involved in pattern separation performance but not FNAME. As a *post-hoc*, exploratory analysis, we analyzed three tasks of executive functioning also completed by these same participants that measure set-shifting (Number-Letter), inhibition (Flanker), and working memory (Keep Track; see Glisky et al., 2021; Ryan et al., 2025). In the Number-Letter task, participants switched between responding even/odd or consonant/vowel to pairs of numbers and letters (e.g., 8E). The Flanker task required participants to respond to the direction of a middle arrow for congruent (< < < < < ) and incongruent trials (< < > < < ). Finally, Keep Track is a working memory task where participants recalled the most recent items presented within a semantic category (e.g., sports, fruits, furniture, etc.). Additional procedures about the executive functioning tasks and measures can be found under Tests of Executive Functions within the Supplementary Section. Respective outcome measures from the Number-Letter, Flanker, and Keep Track tasks included global shift cost, Flanker effect, and percent correct across all trials. Cortical gray matter perfusion was correlated with the Flanker effect (i.e., the primary outcome for the Flanker task; *r* = −0.28, *p* < 0.05; Figure 4), but not with outcomes from the other two executive functioning tasks. Furthermore, the Flanker effect was correlated with pattern separation (*r* = −0.23, *p* < 0.05) but not FNN (*r* = 0.01).

### 3.4 Regional analysis

One-sided *t*-tests to assess gray matter perfusion differences in the ACA, MCA, and PCA between the controls who did not report having a diagnosis of COVID-19 and each COVID-19 group revealed lower perfusion in the hospitalized group in each region (ACA perfusion: *t*_(24)_ = 1.75, *p* < 0.05, *d* = 0.74; MCA perfusion: *t*_(24)_ = 1.80, *p* < 0.05, *d* = 0.77; PCA perfusion: *t*_(24)_ = 1.82, *p* < 0.05, *d* = 0.78). One way ANOVAs without the controls indicated a marginal group difference for perfusion in the ACA [*F*_(2, 59)_ = 2.96, *p* = 0.06] and non-significant differences for the MCA [*F*_(2, 59)_ = 2.23, ns] and PCA [*F*_(2, 59)_ = 2.31, *p* = 0.06] The hospitalized group had lower gray matter perfusion compared to those with respiratory symptoms (ACA perfusion: *t*_(32)_ = 2.34, *p* < 0.05, *d* = 0.95; MCA perfusion: *t*_(32)_ = 2.00, *p* < 0.05, *d* = 0.80; PCA perfusion: *t*_(32)_ = 2.19, *p* < 0.05, *d* = 0.89) and those without respiratory symptoms in the ACA [*t*_(32)_ = 1.71, *p* < 0.05, *d* = 0.69] and PCA [*t*_(32)_ = 1.98, *p* < 0.05, *d* = 0.80] (perfusion differences in MCA were marginal, *t*_(32)_ = 1.60, *p* = 0.06, *d* = 0.65). The groups with and without respiratory symptoms did not differ in any of the three regions, all *t*'s < 1.1, ns. None of the comparisons of white matter perfusion in any arterial region between controls and the COVID-19 groups were significant (*t*'s < 1.42, ns), and similarly, none of the comparisons between the COVID-19 groups were significant (*t*'s < 1.57, ns).

### 3.5 Diffusion results

Group means (SD) for global FA, MD, RD, and AD are displayed in Table 5. Each severity group was compared to the control group who did not report a diagnosis of COVID-19 using independent samples *t*-tests. No differences between controls and any of the COVID-19 groups for any global measure were significant, all *t*'s < 1.2 One-way ANOVAs between the three COVID-19 groups were used to assess the effect of respiratory severity, and no differences were found, FA [*F*_(2, 58)_ = 0.57, ns], MD [*F*_(2, 58)_ = 0.39, ns], RD [*F*_(2, 58)_ = 0.41, ns], or AD [*F*_(2, 58)_ = 0.40, ns]. Correlations between age, global measures of diffusion (controlled for age) and primary cognitive measures revealed no significant relationships.

**Table 5:** Average global FA, MD, AD, and RD across the COVID-19 severity groups.

Group	Global FA	Global MD	Global AD	Global RD
Controls (*N* = 18)	0.42 (0.02)	0.00040 (0.000023)	0.00059 (0.000038)	0.00030 (0.000017)
No respiratory symptoms (*N* = 25)	0.42 (0.02)	0.00041 (0.000023)	0.00061 (0.000036)	0.00031 (0.000018)
With respiratory symptoms (*N* = 26)	0.42 (0.02)	0.00040 (0.000024)	0.00060 (0.000037)	0.00031 (0.000019)
Hospitalized (*N* = 8)	0.42 (0.01)	0.00041 (0.000011)	0.00060 (0.000015)	0.00031 (0.000009)

No differences between controls and each COVID-19 group or between all COVID-19 groups were found.

## 4 Discussion

To summarize the main results, the severity of respiratory symptoms did not significantly impact performance on tests of hippocampal functioning following COVID-19 infection. However, lower gray perfusion was observed among the hospitalized group compared to all other groups, and similar results were found among the three arterial territories. Despite cognitive performance being insignificant at the group-level, a unique relationship between gray matter perfusion and pattern separation was found across all participants. The perfusion results are discussed first, followed by the findings from the cognitive tests and its relationship with perfusion.

### 4.1 COVID-19 severity and perfusion differences: individual differences

Poorer gray matter perfusion existed in the COVID-19 cases who were hospitalized with severe respiratory symptoms compared to controls who did not report a diagnosis of COVID-19 and both non-hospitalized groups over a year from infection. The low sample size of the hospitalized group may have weakened the statistical power in the analyses, warranting caution with interpretation, though the magnitude of effect sizes suggests that these findings may be large enough to detect within our sample. Findings presented here also align with other studies that demonstrated poorer brain perfusion in severe, hospitalized cases using shorter recovery times (Helms et al., 2020; Chougar et al., 2020; Ardellier et al., 2023). This suggests the persistence of perfusion dysfunction following infection among those who were hospitalized, even after a year. It is possible that poorer perfusion in the current study may be explained simply by being hospitalized, given there was no control group of hospitalized non-COVID-19 participants. However, a previous study that divided hospitalized individuals into mild and severe cases found perfusion differences only in the severe group when compared to controls (Qin et al., 2021), suggesting that perfusion abnormalities are not always among hospitalized participants and may vary based on the severity of illness, not necessarily from hospitalization. Additionally, some evidence exists to suggest that perfusion changes from COVID-19 begin to recover over time, but not completely (Tian et al., 2022). The same participants from Qin et al. (2021) were evaluated at 10 months post-infection in a separate study, and they found that severe cases showed overall improvements in cortical CBF compared to cortical CBF measured at 3 months. However, this was not a full recovery, as severe cases still demonstrated hypoperfusion in the left insula compared to the three-month timepoint. Additionally, a comparison between severe cases and controls without reported COVID-19 indicated that severe cases still had lower CBF, particularly in frontal and temporal cortices (Tian et al., 2022). Continuing to use longer time points will be critical to better understand the extent of perfusion recovery.

A large amount of variability was found in the non-hospitalized COVID-19 groups, likely making it difficult to detect mean differences compared to controls. The heterogeneity in these groups suggests that some, but not others, are experiencing hypoperfusion in the gray matter. Some individuals without respiratory symptoms had higher gray matter perfusion than controls, while others had comparable perfusion estimates to those who were hospitalized. Few studies have investigated perfusion differences between controls and those with COVID-19 exclusively in a non-hospitalized sample. Lower gray matter CBF was found in non-hospitalized cases compared to controls using on average four (Kim et al., 2023) and 6 months from infection (Ajčević et al., 2023). The longer recovery time in the present study might have been sufficient for perfusion levels to return to baseline among some participants with less severe symptoms, which has been previously shown to occur following 10 months in a longitudinal study (Tian et al., 2022). The broad range of perfusion levels across all groups highlights individual differences and that the respiratory symptoms during the acute infection may not be a strong predictor of brain perfusion dysfunction, particularly in non-hospitalized cases.

The data presented here do not provide strong evidence for regional specificity, but rather more global perfusion differences from COVID-19. The cortical gray matter in the ACA, MCA, and PCA mirrored the results from the total cortical gray matter perfusion, with only the hospitalized group demonstrating poorer perfusion within the total cortical gray matter and within arterial territories. Decreased brain perfusion has been suggested to be linked to more global rather than regional changes (Ardellier et al., 2023). For example, in a study with non-hospitalized participants 6 months from infection, Ajčević et al. (2023) found lower total cortical gray matter perfusion compared to controls, and also demonstrated lower perfusion in the frontal, parietal, and temporal lobes. We divided regions based on the arterial territories rather than anatomical regions, however, the current results are still largely replicated. With the exception of the occipital lobe, which is perfused by both the MCA and PCA, all of the anatomical regions from Ajčević et al. (2023) were within arterial territories that had lower perfusion in the present study.

Perfusion abnormalities in COVID-19 can be linked to several possibilities, including BBB dysfunction. SARS-CoV-2 binding to the ACE2 receptors along the vascular endothelium can alter the permeability of the BBB (Buzhdygan et al., 2020; de Sousa et al., 2020). Additionally, independent and interacting effects of inflammation are likely involved with perfusion changes in post-COVID. Heightened inflammation is a hallmark of COVID-19 (Heneka et al., 2020; Mehta et al., 2020; Aghagoli et al., 2021; Wang et al., 2020), and other conditions known to increase inflammation have shown damage to the BBB (Cheng et al., 2018; Sharshar et al., 2007). Finally, pericytes wrapping the endothelium are a primary site for SARS-CoV-2 and ACE2 binding in the brain (Muhl et al., 2022; Bocci et al., 2021), resulting in vasoconstriction and decreased CBF (Hirunpattarasilp et al., 2023).

Many previous studies of perfusion with COVID-19 participants report perfusion levels in the gray matter only. One previous study also evaluated white matter perfusion among non-hospitalized post-COVID participants and did not find differences between controls as well (Fineschi et al., 2024). It is important to note that gray matter perfusion is suggested to be the gold standard brain perfusion metric in future studies (Chappell et al., 2021). Cortical gray matter perfusion was lower among those with severe respiratory symptoms and sensitive to pattern separation, but these relationships did not exist for white matter perfusion. Consistent with this, analysis of white matter integrity using DTI also revealed no differences between controls and COVID-19 groups. Furthermore, no measure of diffusion was correlated with any cognitive outcomes. This suggests that disrupted perfusion may be more characteristic of long-term COVID-19, while damage to the white matter integrity is not. One longitudinal study also evaluating both diffusion and perfusion outcomes after 3 and 10 months from infection among hospitalized cases has comparable findings (Tian et al., 2022). At 3 months, severe hospitalized participants demonstrated lower FA in multiple white matter tracts and also reduced cortical CBF in the gray matter compared to controls. Perfusion and diffusion outcomes tended to improve after 10 months. However, CBF was still lower than controls while FA values in all tracts were no longer different compared to controls, after correcting for multiple comparisons. On the other hand, other studies indeed find prolonged damage to the white matter. For example, Huang et al. (2022) found lower FA in the body of the corpus callosum among ICU COVID-19 participants compared to non-ICU COVID-19 participants a year from infection, and lower global FA was observed among those diagnosed with PCS 11 months from infection (Díez-Cirarda et al., 2023a). It is important to note that the participants in the current study ranged in respiratory symptom severity and most were non-hospitalized, regardless of whether they reported ongoing cognitive symptoms. Additionally, analysis of white matter integrity was conducted at a global level in the current study, which may not be sensitive to potential changes occurring within specific tracts. These discrepancies may explain the conflicting results.

### 4.2 Perfusion and hippocampal task outcomes

Studies of COVID-19 and the impact directly on the hippocampus are seldom. However, one study found that those with post-COVID syndrome had smaller hippocampal volumes than healthy controls after 11 months, and this was the most pronounced in hospitalized cases (Díez-Cirarda et al., 2023b). This has implications for performance on pattern separation tasks, given smaller hippocampal volumes are correlated with pattern separation performance on the MST (Doxey and Kirwan, 2015). Pattern separation is particularly sensitive to the structural and functional integrity of the hippocampus in aging (Stark et al., 2013, 2015; Yassa et al., 2011; Dillon et al., 2017; Holden et al., 2013; Toner et al., 2009; Stark and Stark, 2017; Doxey and Kirwan, 2015). Additionally, performance on FNAME has also been shown to rely on the integrity of the hippocampus, suggesting that associative-memory performance may also be particularly vulnerable to poor performance following COVID-19 (Papp et al., 2014; Sperling et al., 2003).

The current study indicated that the hospitalized group tended to perform worse on pattern separation and recall of names, though this did not reach statistical significance. There are several explanations that could support these findings. First, potentially the effect is subtle and requires larger samples to detect. Second, COVID-19 may not particularly impact hippocampal functioning, but rather result in broad impairment across many domains. A recent review and meta-analysis including studies of up to a year following infection concluded a lack of a consistent pattern of cognitive dysfunction in the literature (Fanshawe et al., 2025), emphasizing the significant variability in the literature. Third, cognitive dysfunction may exist early on, but begin to recover after longer recovery times. For example, one study demonstrated poorer pattern separation at an earlier timepoint of 6 months (Zhao et al., 2022). They used a computerized object memory task 6 months following infection among younger adults who had mild cases of COVID-19 (*n* = 80), and found that COVID-19 participants had greater difficulty recalling the correct object in the midst of other highly similar objects differing in orientation and other subtle details compared to controls. Recovery in cognitive performance over time would be relatively consistent with some studies evaluating cognitive performance after 2 months (Peskar et al., 2023) and beyond 6 months (Nersesjan et al., 2022; Ferrucci et al., 2022). The meta-analysis from Fanshawe et al. (2025) found that many other longitudinal studies do not show a change over time. However, no study included in their meta-analysis used a recovery timepoint beyond a year, making the current study a critical step forward to evaluate cognitive functioning at longer recovery times.

In the current study, gray matter perfusion predicted pattern separation performance for both controls who did not report having COVID-19 and those with COVID-19, such that better performance was associated with greater levels of cortical perfusion. We did not have the level of specificity to evaluate perfusion directly in the hippocampus. However, only one study to our knowledge has investigated pattern separation with the MST and perfusion within the hippocampus using ASL (Memel et al., 2021). In this study of cognitively normal older adults, gray matter CBF in the hippocampus was moderated by e4 status. Non-carriers demonstrated a positive relationship between pattern separation and perfusion, while carriers demonstrated the opposite relationship, whereby greater perfusion was associated with poorer performance. In the present study, hypoperfusion was observed among the hospitalized group, which makes it somewhat surprising that they did not perform statistically worse than the other groups on correctly recalling objects, though the directionality was still in the expected direction.

Gray matter perfusion related to pattern separation scores but not with FNN. Given that both tasks are known to rely on the hippocampus, one might expect that if perfusion abnormalities were predominantly affecting the hippocampus, then both tasks would show similar associations with perfusion. However, the data in the present study suggest otherwise, indicating that perfusion abnormalities associated with COVID-19 may not be specific to the hippocampus. This finding is in contrast to a study done by Díez-Cirarda et al. (2023b), which found evidence for hippocampal perfusion abnormalities using a similar sample size. A few differences in their study may explain the discrepant results. First, their sample focused on individuals who had a diagnosis of post-COVID syndrome (PCS), possibly excluding individuals with subtle COVID-19 changes. In a *post-hoc* analysis assessing cortical gray matter perfusion between those with COVID-19 who reported ongoing cognitive symptoms and those who did not, we found no differences in cortical gray matter perfusion, suggesting that hypoperfusion is not exclusive to those who report ongoing symptoms. This provides some initial insight into the relationship between subjective cognitive decline and cerebral perfusion, but future studies using more thorough testing to assess subjective decline are clearly needed. Second, their analyses found a difference between their non-hospitalized and hospitalized groups, which is largely replicated here. Unfortunately, no analysis comparing their non-hospitalized and healthy controls was conducted, making it unclear how perfusion compared between milder cases and controls. Finally, their study focused only on hippocampal perfusion, leaving ambiguity about whether perfusion abnormalities were reflecting global or specific changes. Given that the results from the three arterial territories were highly correlated with the total perfusion, the results from Díez-Cirarda et al. (2023b) may be reflecting a global change rather than something specific to the hippocampus.

Other executive functioning processes that are not reliant on the hippocampus may explain why pattern separation, but not FNN, was related to perfusion.

Our exploratory analyses on three executive functioning tasks indicated that the inhibition, as measured by the Flanker task, was uniquely related to both gray matter perfusion and pattern separation. Viewing a similar object in pattern separation with a high degree of overlap would also require one to inhibit the more automatic responses of identifying it as old. Poor inhibition has been suggested as a mechanism that impacts memory encoding and retrieval in aging that results in “hyperbinding” (Amer et al., 2022; Campbell et al., 2010; Campbell and Hasher, 2018), making tasks such as pattern separation more difficult for older adults. However, this framework in aging is debated (Palmer et al., 2022). Nonetheless, executive functioning using composite scores and MST performance have been shown to be correlated in recent studies, but none specifically evaluated the role of inhibition separately (Jensen et al., 2023; Gellersen et al., 2021). Interestingly, the meta-analysis from Fanshawe et al. (2025) evaluated prognostic factors for domain-specific impairments and found respiratory symptom severity to be the best predictor, correlating with executive dysfunction and complex attention. This would be relatively consistent with the current study showing that severe respiratory symptoms among the hospitalized group tended to be associated with the worst memory performance, potentially due to executive dysfunction.

### 4.3 Conclusions

The connection between cerebral vascular dysfunction and COVID-19 has been evident since the start of the pandemic. However, we are still discovering how cerebrovascular changes can predict long-COVID sequelae, particularly cognitive impairments. The most severe cases of COVID-19 demonstrated lower gray matter perfusion, but individual differences were also apparent in less severe cases. Future studies with larger sample sizes will continue to be a need for ongoing research. Gray matter perfusion also uniquely predicted pattern separation performance, but this may be related to more executive dysfunction following infection rather than impairment specific to the hippocampus. Additionally, the lack of prolonged damage to the white matter provides further support that disrupted perfusion is at least one of the prominent drivers of cognitive sequelae among severe COVID-19 cases over a year from infection.

## Data Availability

The raw data supporting the conclusions of this article will be made available by the authors, without undue reservation.
