# Supplementary material for: Reduced cortical brain perfusion following COVID-19 infection: impact of COVID-19 severity and relation to memory performance
Source: Front Hum Neurosci. 2026 Apr 10;20:1575787. doi: 10.3389/fnhum.2026.1575787 (PMC13106290; doi:10.3389/fnhum.2026.1575787)
Supplement: Supplementary file 1 [file Data_Sheet_1.docx]

Supplementary Material

*Correcting for PVE*

Partial volume corrected gray and white matter perfusion values were calculated within a binary cortical mask adapted from Liu et al. (2023) (see **Supplementary** **Figure 1**). To ensure that the approach to identifying gray matter was not significantly impacting perfusion values, we correlated perfusion values using three different methods to correct for PVE (**Supplementary** **Table 1**): 1) whole-brain gray matter thresholded at 50%, 2) whole-brain gray matter, unthresholded, and 3) cortical gray matter using our custom mask, unthresholded. All three methods used PVC. It is clear to see that these values are highly correlated and the choice of these metrics will likely not alter results.


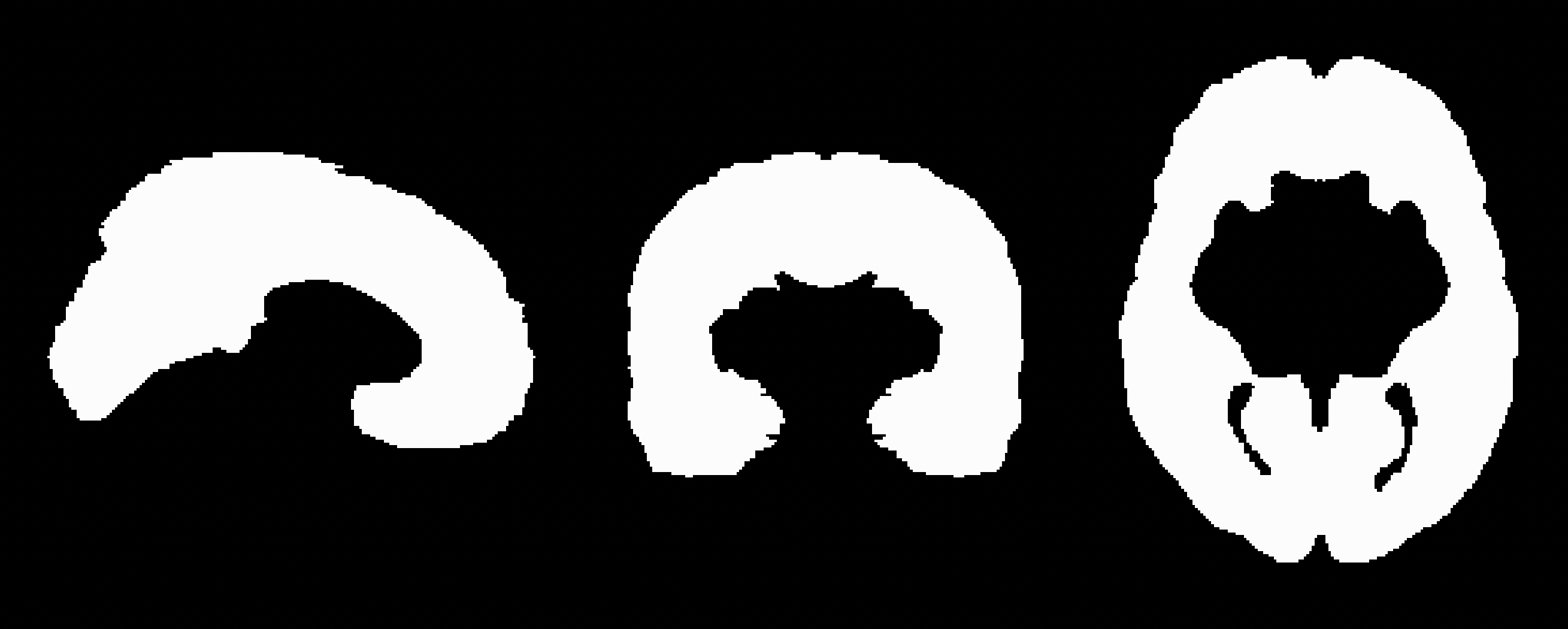


**Supplementary Figure 1**: Binary cortical mask created from Liu et al. (2023) that will be used to calculate gray and white matter cortical perfusion.

|  | **Total brain GM at 50 + pv corrected** | **Total brain GM pv corrected only** | **Cortical GM pv corrected** |
| --- | --- | --- | --- |
| Total brain GM at 50 + pv corrected | 1 |  |  |
| Total brain GM pv corrected only | 0.999** | 1 |  |
| Cortical GM pv corrected | 0.996** | 0.998** | 1 |

**Supplementary Table 1:** Correlation matrix between the three different approaches to measure gray matter perfusion. Measures are highly correlated and will not likely impact subsequent results. ** indicates p<0.01

*Creation of the Arterial Territory Mask*

We multiplied the Liu et al. (2023) ArterialAtlas_level2 mask with the above binary cortical mask, producing a cortical mask with the anterior cerebral artery (ACA), middle cerebral artery (MCA), and posterior cerebral artery (PCA) labeled. Left and right regions within each territory were combined due to the exceptionally high correlations (**Supplementary** **Table 2**).

| **GM** | **Left ACA** | **Right ACA** | **Left MCA** | **Right MCA** | **Left PCA** | **Right PCA** |
| --- | --- | --- | --- | --- | --- | --- |
| Left ACA |  |  |  |  |  |  |
| Right ACA | .980** |  |  |  |  |  |
| Left MCA | .937** | .911** |  |  |  |  |
| Right MCA | .913** | .933** | .922** |  |  |  |
| Left PCA | .719** | .711** | .798** | .773** |  |  |
| Right PCA | .731** | .749 | .779** | .812** | .906** |  |

**Supplementary Table 2:** Correlation matrix between the left and right arterial territories (highlighted). Left and right regions are highly correlated and were combined for all other analyses. ** indicates p<0.01.


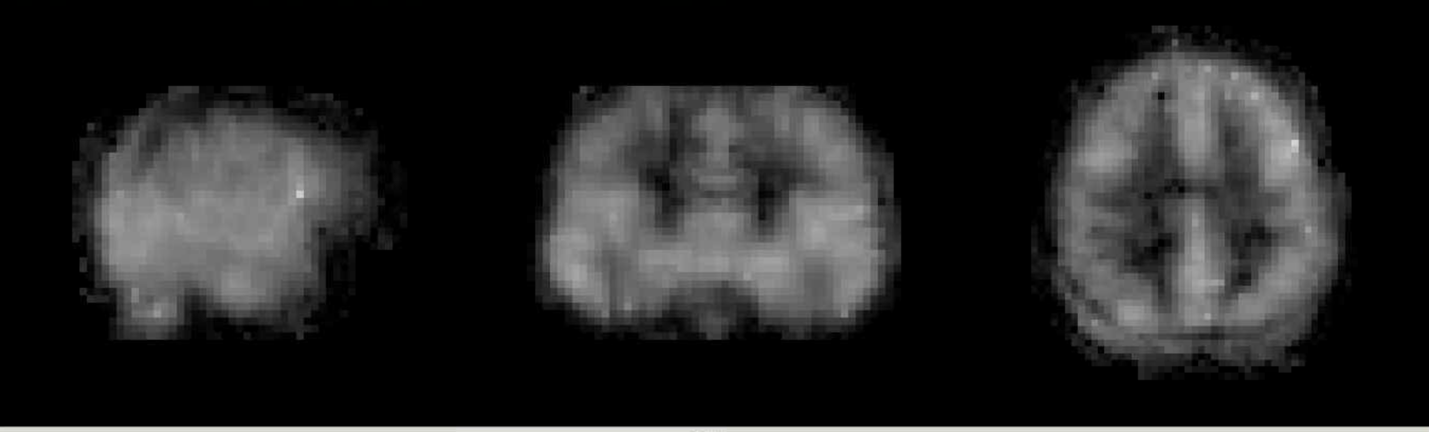


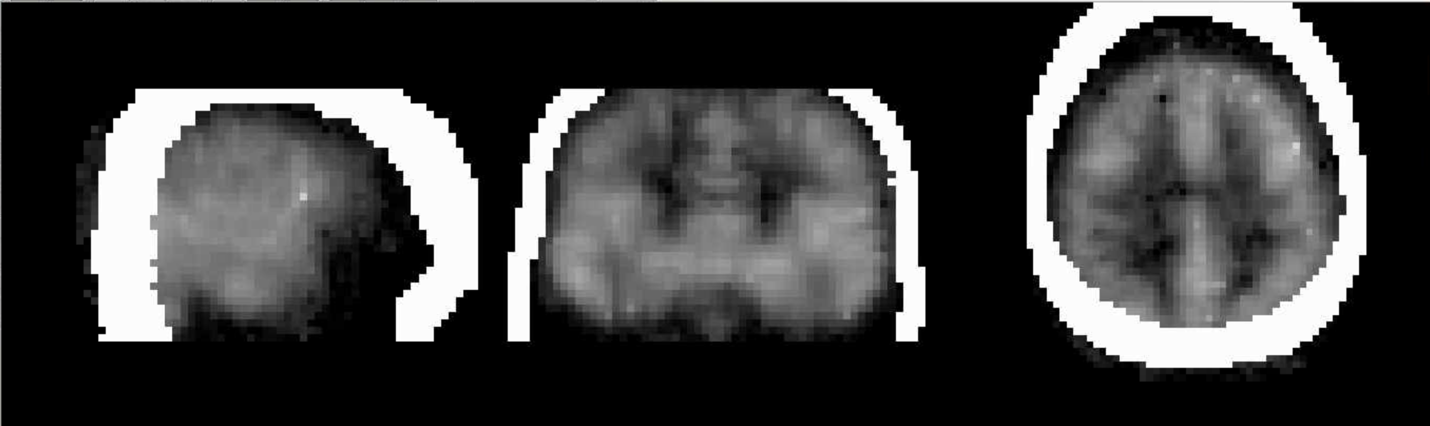


**Supplementary Figure 2**: Top: Example of chemical shift observed in posterior region of participant’s CBF map. Bottom: Calculated mask used to ignore potentially corrupt voxels.

Tests of Executive Functions

**Number-Letter:** Number-Letter assesses switching (Glisky et al., 2021). This task was presented on the computer using E-Prime. A number-letter pair (e.g., 7G) was presented in one of four quadrants on the computer screen. When the number-letter pair appeared in the top quadrants, participants identified if the number was odd or even using the “1” and “2” keys on the keyboard with their right hand. When the number-letter pair appeared in the bottom quadrants, participants identified if the letter was a consonant or vowel using the “C” and “V” keys with their left hand. The first block consisted of 32 trials where the number-letter pair appeared only on the top half of the screen, requiring participants to only respond to the number. The second block also consisted of 32 trials, where the number-letter pair appeared only on the bottom, requiring participants to respond to only the letter. Ten practice trials preceded each of these two blocks. In the final block of 64 trials (known as the shift block), the number-letter pair moved in a clockwise fashion across trials starting in the top left, requiring participants to shift between responding to the numbers (top half of the screen) and letters (bottom half of the screen). There was a 150 ms ITI. Twelve practice trials preceded this final block. The dependent measure was the global shift cost calculated as the difference between the average reaction time (ms) for the shift block and the average reaction time (ms) across the first two non-shift blocks.

**Flanker:** The Flanker is a task that requires participants to sustain attention on one aspect of the stimulus while ignoring the surrounding components, often referred to as inhibition (Eriksen & Eriksen, 1974). This task was presented on the computer using PsyToolkit. Participants were shown a row of five arrows in the center of the computer screen and instructed to respond to the arrow in the middle. If the middle arrow was pointing to the left (←), participants pressed the “A” key, and if the middle arrow was pointing to the right (→), participants pressed the “L” key on the keyboard. Participants were given 50 trials in a fixed order. Half of the trials showed all five arrows pointing the same direction (congruent) and half of the trials showed the middle arrow pointing in the opposite direction of all the other arrows (incongruent). Each trial was on the screen 500 ms, with an ITI of 300 ms. Eight practice trials with feedback were provided before the start of the task. The dependent measure was the reaction time (ms) for correct responses on the incongruent trials subtracted by the reaction time (ms) for correct responses on congruent trials, known as the Flanker effect.

**Keep Track:** Keep Track is a working memory task that requires participants to remember the most recent items from various categories (Glisky et al., 2021). This task was completed on the computer using E-Prime. Participants were shown a series of 15 words belonging to one of six categories (relatives, metals, sports, furniture, fruits, and distances) and asked to keep track of the last word presented from several of these categories. Each word was shown for 1500 ms, 150 ms ITI. Prior to the start of the task, participants were shown each of the 36 words used in the task (six words per category), one at a time, in the center of the computer screen, and identified the category to which the word belonged. An example and practice trial were given prior to the start of the test trials. For the test trials, participants were required to remember the last word from one, two, three, and then four categories. The names of the target categories were at the bottom of the screen throughout the trial. Three lists were presented for each category size, resulting in 30 total trials with increasing working memory load. The dependent measures were the percent of correct responses for each category size.
